# Microsomal Prostaglandin E Synthase-1 Controls Colonic Prostaglandin E_2_ Production and Exerts a Protective Effect on Colitis Induced by Trinitrobenzene Sulfonic Acid in Mice

**DOI:** 10.3390/ijms252212326

**Published:** 2024-11-17

**Authors:** Fumiaki Kojima, Yuka Hioki, Hiroki Sekiya, Hitoshi Kashiwagi, Yoshiko Iizuka, Kei Eto, Shotaro Maehana, Fumitaka Kawakami, Makoto Kubo, Hitoshi Ishibashi, Takafumi Ichikawa

**Affiliations:** 1 Department of Pharmacology, Kitasato University School of Allied Health Sciences, Sagamihara 252-0373, Japan; 2 Department of Regulation Biochemistry, Kitasato University Graduate School of Medical Sciences, Sagamihara 252-0373, Japan; kawakami@kitasato-u.ac.jp (F.K.); t.ichika@kitasato-u.ac.jp (T.I.); 3 Regenerative Medicine and Cell Design Research Facility, Sagamihara 252-0373, Japan; yiizuka@kitasato-u.ac.jp (Y.I.); keieto@kitasato-u.ac.jp (K.E.); smaehana@kitasato-u.ac.jp (S.M.); kuboma@kitasato-u.ac.jp (M.K.); isibasih@kitasato-u.ac.jp (H.I.); 4 Faculty of Pharmaceutical Sciences, Hokkaido University, Sapporo 060-0812, Japan; h-kashi@pharm.hokudai.ac.jp; 5 Department of Public Health, Kitasato University Graduate School of Medical Sciences, Sagamihara 252-0373, Japan; 6 Department of Physiology, Kitasato University School of Allied Health Sciences, Sagamihara 252-0373, Japan; 7 Department of Environmental Microbiology, Kitasato University Graduate School of Medical Sciences, Sagamihara 252-0373, Japan

**Keywords:** prostaglandin E synthase, cyclooxygenase, prostaglandin E_2_, inflammatory bowel disease, colitis, Th17 and Th1 cytokines

## Abstract

Microsomal prostaglandin E synthase-1 (mPGES-1) is an isozyme of the prostaglandin (PG) E synthase that acts downstream of cyclooxygenase and catalyzes the conversion of PGH_2_ to PGE_2_. The impact of genetic deletion of mPGES-1 on the development of 2,4,6-trinitrobenzene sulfonic acid (TNBS)-induced colitis, a well-established model of inflammatory bowel disease (IBD), was investigated in this study. After administration of TNBS, mice deficient in mPGES-1 (mPGES-1^−/−^ mice) showed more severe colitis than did wild-type (WT) mice. Histological examination revealed that mPGES-1^−/−^ mice had markedly exacerbated symptoms of colitis. mPGES-1 expression was detectable in the colons of WT mice at both the mRNA and protein levels. Lack of mPGES-1 resulted in marked reduction of colonic PGE_2_ production. Our study also showed a significant increase in colonic expression of interleukin-17A (IL-17A), as well as interferon γ (IFNγ) and tumor necrosis factor α, during colitis in mPGES-1^−/−^ mice compared with that in WT mice. Furthermore, loss of mPGES-1 increased the populations of IL-17A-producing T-helper (Th) 17 and IFNγ-producing Th1 cells in mesenteric lymph nodes. These results suggest that mPGES-1 is the main enzyme responsible for colonic PGE_2_ production and deficiency of mPGES-1 facilitates the development of colitis and T-cell-mediated immunity. mPGES-1 might, therefore, impact T-cell-related immune response associated with IBD.

## 1. Introduction

Inflammatory bowel disease (IBD) is a chronic inflammatory condition characterized by abnormalities of the immune system. The two primary disease categories of IBD are ulcerative colitis and Crohn’s disease. T cells, which differentiate into various subsets of T-helper (Th) cells, such as Th1, Th2, Th17, and regulatory T cells (Tregs), in response to different cytokines, are well known to play a significant role in the pathogenesis of IBD [1]. The progression of IBD is closely associated to the cytokine network and aberrant immune responses. Monoclonal antibodies targeting Th1 and Th17 cytokines have led to the development of biological therapies, some of which have shown clinical effectiveness in treating IBD [2].

Prostaglandin (PG) E_2_ functions as a lipid mediator that plays a crucial role in various physiological and pathological processes. The synthesis of PGE_2_ is controlled by a series of enzymatic reactions, primarily involving cyclooxygenase (COX) and PGE synthase (PGES) [3,4]. Previous studies have identified at least three PGES isozymes, including cytosolic PGES (cPGES), microsomal PGES-1 (mPGES-1), and mPGES-2, that are involved in the final phase of PGE_2_ biosynthesis following COX activity [5,6,7,8]. PGE_2_ is known to be produced in elevated amounts in the inflamed mucosa of IBD patients [9]. An analysis of biopsies from IBD patients demonstrated that both PGE_2_ production and COX-2 expression increase during the active stages of the disease [10]. Numerous studies have suggested non-steroidal anti-inflammatory drugs (NSAIDs), which block the enzymatic activity of COX, to treat the onset or aggravation of IBD [11].

A subtype of PGES, mPGES-1, specifically catalyzes the conversion of PGH_2_ to PGE_2_ in various cell types and tissues [5,6]. A previous study indicated that mPGES-1 is highly expressed in the inflamed intestinal mucosa of patients with Crohn’s disease and ulcerative colitis [12], implying the significance of mPGES-1 in the development of IBD. Various studies conducted on mice deficient in mPGES-1 (mPGES-1^−/−^ mice) has yielded several new insights into the potential function of mPGES-1 as a crucial mediator in various physiological and pathophysiological processes associated with inflammation and immune response [13,14,15,16,17,18,19,20,21,22]. It has been shown by us and others that mPGES-1^−/−^ mice exhibit increased susceptibility to dextran sulfate sodium (DSS)-induced colitis [23,24,25], although the precise intrinsic mechanisms underlying this susceptibility have yet to be fully understood.

The hapten reagent 2,4,6-trinitrobenzene sulfonic acid (TNBS)-induced colitis model is widely used as a well-established model of IBD and is known to induce a T-cell-dependent immunogenic reaction, mainly mediated by Th17 and Th1 cells in the colon [26,27]. In particular, the pathogenesis of Crohn’s disease is thought to be driven by Th1-mediated responses, while Th2-mediated responses are believed to be associated with ulcerative colitis [28]. In addition to Th1-mediated responses, recent evidence indicates that Th17-mediated responses significantly accelerate the progression of Crohn’s disease. Previous research has shown that the levels of Th1 cytokines, such as interferon-γ (IFNγ) and interleukin (IL)-12, along with Th17 cytokines, including IL-17A and IL-23, are markedly elevated in Crohn’s disease and in mouse colitis models including TNBS-induced colitis, which is a relevant model for studying Crohn’s disease [29,30,31,32]. In the present study, we aimed to evaluate the impact of mPGES-1 deletion on the biosynthesis of PGE_2_ in the colon and on the development of TNBS-induced colitis associated with Th17 and Th1 immunological responses in mice.

## 2. Results

### 2.1. Exacerbated Colitis in mPGES-1^−/−^ Mice

**Figure 1:**
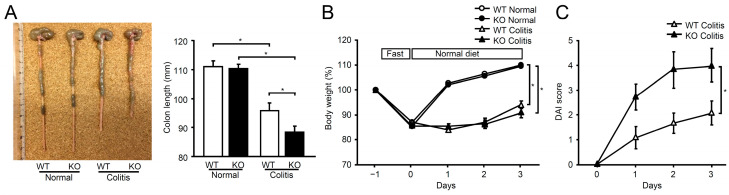
Impact of mPGES-1 genetic deletion in TNBS-induced colitis. (**A**) On day 3 following TNBS administration, the length of each colon was assessed as an indirect indicator of inflammation (*n* = 11 to 13). Colon photographs are typical examples in WT and mPGES-1^−/−^ mice. (**B**) Time course of body weight changes in WT and mPGES-1^−/−^ mice before and after TNBS administration (*n* = 17); Mice were fasted overnight and then TNBS was administered on day 0; (**C**) The progression of DAI scores after the specified days of TNBS administration (*n* = 11 to 12). *, *p* < 0.05; ANOVA followed by the Bonferroni test.

mPGES-1^−/−^ mice and wild-type (WT) mice were administered an enema of 2% TNBS in 40% ethanol, and the severity of colitis was evaluated over a period of 3 days. It has been demonstrated that colon shortening serves as a valuable inflammatory marker and indicator of colitis [33]. On day 3, colon shortening was observed in both genotypes of TNBS-treated mice, with mPGES-1^−/−^ mice exhibiting a significantly shorter colon than WT mice (Figure 1A). In both genotypes, TNBS-treated mice showed a marked reduction in body weight compared to normal mice; however, no significant difference was noted between WT and mPGES-1^−/−^ mice (Figure 1B). The Disease Activity Index (DAI) colitis score, calculated by summing the diarrhea score and fecal bleeding score, significantly increased in mPGES-1^−/−^ mice compared to WT mice. Notably, in our preliminary experiment, only about 70% of mPGES-1^−/−^ mice (8 out of 11) survived by day 4 following TNBS administration, while all WT mice (12 out of 12) survived. Consequently, the endpoint for colitis induction was established on day 3 after TNBS administration in this study.

### 2.2. Histological Characteristics of Colitis in mPGES-1^−/−^ Mice

**Figure 2:**
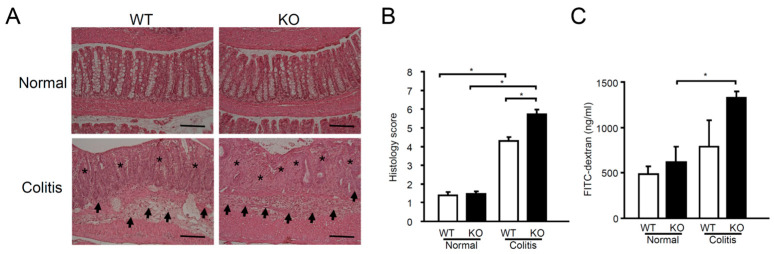
Histological evaluation of TNBS-induced colitis in mPGES-1 deficiency. (**A**) On day 3 after TNBS administration, colon sections of mPGES-1 WT and mPGES-1^−/−^ mice were stained with H&E. Results are typical pictures using the Swiss roll method (*n* = 11 to 17). Stars and arrows in the panel indicate epithelial damage and inflammatory infiltration in mPGES-1^−/−^ mice relative to WT mice. Scale bar represents 100 µm. (**B**) A blinded researcher evaluated the histology scores by calculating the sum of the inflammatory infiltration score and the epithelial damage score, with a maximum possible score of 8 (*n* = 11 to 17). (**C**) On day 3 following the injection of TNBS, intestinal permeability was assessed using FITC-dextran. (*n* = 3 to 6). *, *p* < 0.05; ANOVA followed by the Bonferroni test.

To further assess the severity of colitis, a histological evaluation was conducted on colon sections stained with hematoxylin and eosin (H&E). As shown in Figure 2A, on day 3 following TNBS administration, the colons of mPGES-1^−/−^ mice exhibited more severe features of colitis, including extensive mucosal tissue damage and edema, compared to WT mice. An observer, blinded to the genotypes of the sections, assessed these histological features, revealing that the combined scores for epithelial damage and inflammatory infiltration were significantly elevated in mPGES-1^−/−^ mice relative to WT mice (Figure 2B). In order to evaluate the beneficial function of mPGES-1 in the epithelium of TNBS-administered mice, we analyzed permeability by orally administering fluorescein isothiocyanate (FITC)-dextran and determining its serum concentrations. A significantly larger amount of FITC-dextran that had diffused through the epithelium in mPGES-1^−/−^ mice was observed on day 3 after TNBS administration, suggesting damage of the epithelial barrier function (Figure 2C).

### 2.3. mRNA Expression of PGE_2_ Biosynthetic Enzymes in TNBS-Induced Colitis

**Figure 3:**
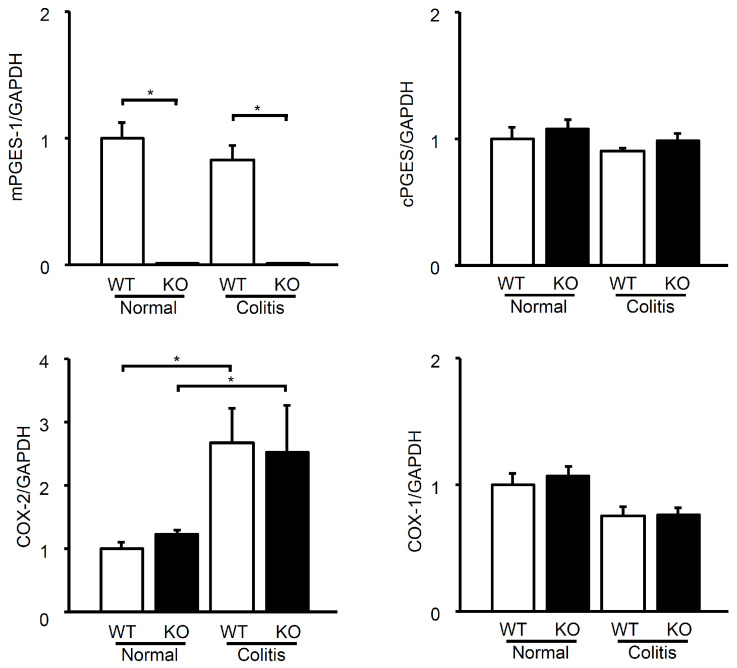
Expression of mRNA for PGE_2_ biosynthetic enzymes in the colon following exposure to TNBS. On day 3 after TNBS administration, the expression levels of mRNA for PGES and COX isozymes in the colons were assessed using real-time RT-PCR. mRNA expression levels are presented as fold induction relative to the expression in WT mice that did not receive TNBS treatment (assigned the value “1”). *, *p* < 0.05; ANOVA followed by the Bonferroni test (*n* = 6).

We then examined the colonic mRNA expression of PGE_2_ synthesis enzymes in WT and mPGES-1^−/−^ mice with or without TNBS treatment, since we found that TNBS-induced colitis was more severe in mPGES-1^−/−^ mice (Figure 3). In WT mice, mPGES-1 mRNA levels in the colon were detectable under basal conditions and did not change by TNBS treatment. Expectedly, mPGES-1 expression was completely absent in mPGES-1^−/−^ mice, regardless of TNBS administration. In both WT and mPGES-1^−/−^ mice, TNBS treatment had no effect on the colonic expression of cPGES mRNA. However, TNBS treatment significantly increased COX-2 mRNA expression in the colons of both WT and mPGES-1^−/−^ mice, with similar expression levels observed in each genotype. COX-1 mRNA was present in the colons of both WT and mPGES-1^−/−^ mice, and its expression level remained unchanged after TNBS administration. These data indicate that the genetic deletion of mPGES-1 does not affect the mRNA expression of its upstream synthase or other PGE isozymes.

### 2.4. Protein Expression of Prostanoid Biosynthetic Enzymes in the Colon

**Figure 4:**
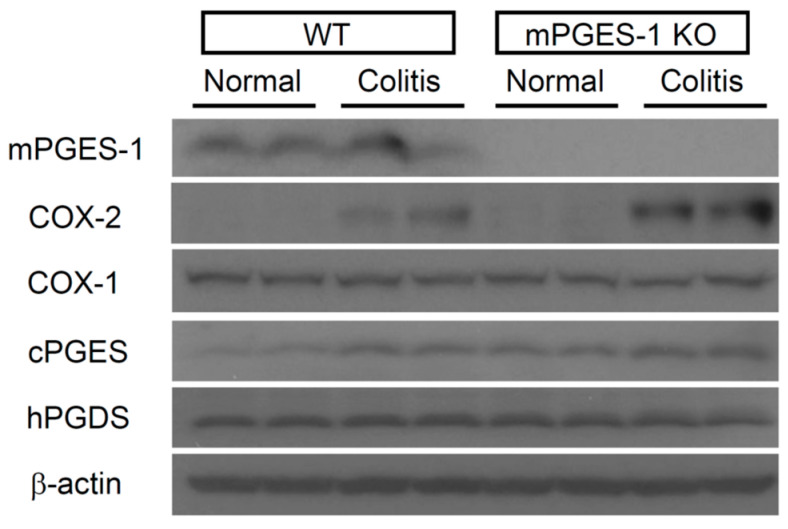
Western blot analysis was conducted to examine the colonic protein expression of PGES, PGDS, and COX on day 3 following TNBS administration (*n* = 3).

Next, we explored the expression levels of proteins related to PGE_2_ biosynthetic enzymes in the colon (Figure 4). mPGES-1 protein was detected in the colon of WT mice, either with or without TNBS administration. Predictably, there was no expression of mPGES-1 protein in the colon of mPGES-1^−/−^ mice. COX-2 protein was induced by TNBS administration in both genotypes. Importantly, the upregulation of COX-2 was more pronounced in mPGES-1^−/−^ mice relative to WT mice. Conversely, cPGES and COX-1 proteins were found in both WT and mPGES-1^−/−^ mice without TNBS administration, and their expression levels remained unchanged following the induction of colitis. No differences in the protein expression of either enzyme were observed between WT and mPGES-1^−/−^ mice. The protein expression profiles of mPGES-1, cPGES, and COX-1 corresponded with their respective mRNA levels. Additionally, hematopoietic PGD synthase (hPGDS) protein was detected in the colons of both WT and mPGES-1^−/−^ mice, with no change in expression levels following colitis induction.

### 2.5. Production of PGE_2_ and PGD_2_ in the Colon

**Figure 5:**
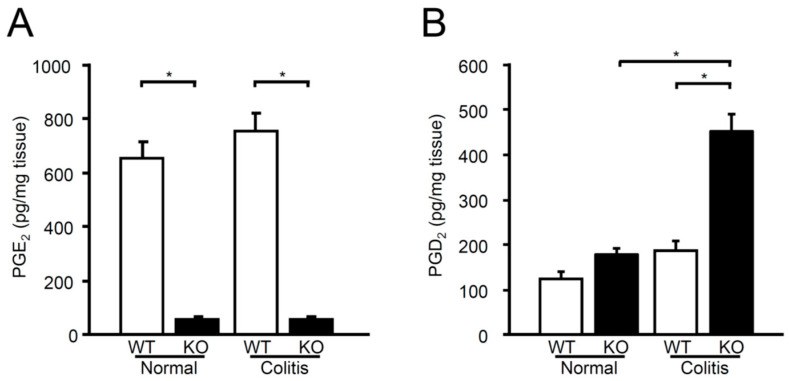
Levels of PGE_2_ (**A**) and PGD_2_ (**B**) in the colons of mice treated or not treated with TNBS were measured using ELISA. *, *p* < 0.05; ANOVA followed by the Bonferroni test (*n* = 6 to 11).

We then evaluated the involvement of mPGES-1 in colonic prostanoid synthesis under both normal and colitis conditions. As shown in Figure 5A, PGE_2_ levels were significantly elevated in the colons of WT mice, regardless of whether TNBS was administered. Importantly, the genetic deletion of mPGES-1, with or without TNBS treatment, led to a greater reduction in colonic PGE_2_ levels compared to WT mice. These findings suggest that mPGES-1 might be primarily responsible for colonic PGE_2_ production in both colitis and healthy states.

In order to assess whether arachidonic acid was being redirected into an alternative prostanoid pathway in mPGES-1^−/−^ mice, we quantified the colonic levels of PGD_2_, which is recognized for its anti-colitis properties [10,34]. WT and mPGES-1^−/−^ mice showed comparable baseline PGD_2_ levels in the colon; however, following TNBS administration, mPGES-1^−/−^ mice exhibited a significant increase in colonic PGD_2_ production, in contrast to WT mice (Figure 5B).

### 2.6. Promotion of Th17/Th1-Related Cytokine Expression in mPGES-1^−/−^ Mice

**Figure 6:**
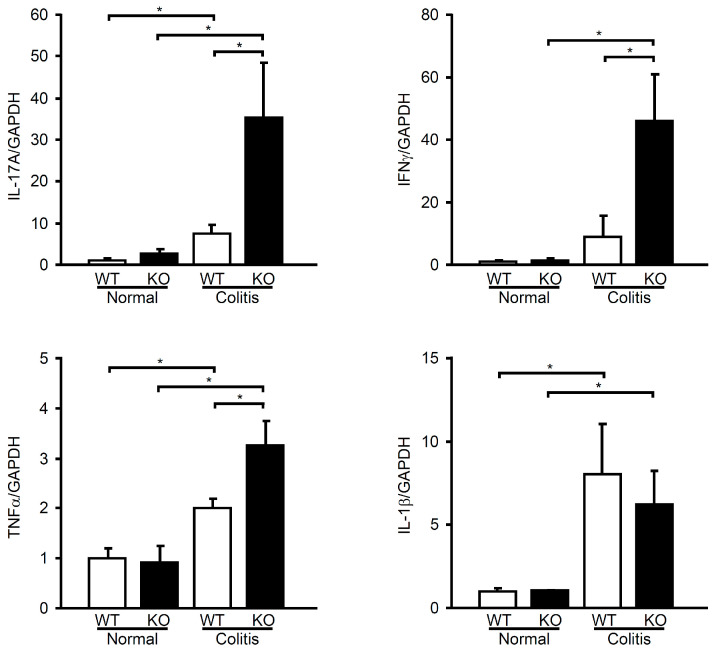
Expression of Th17/Th1-related cytokines in the colon of mPGES-1^−/−^ mice. On day 3 following TNBS administration, the expression of IL-17A, IFNγ, TNFα, and IL-1β mRNA in the colons of mice was examined using real-time RT-PCR. The expression levels are presented as fold changes compared to the expression in WT mice that did not receive TNBS treatment (assigned the value “1”). *, *p* < 0.05; ANOVA followed by the Bonferroni test (*n* = 6).

To elucidate how mPGES-1 produces an anti-colitis effect, we focused on T cell immunity, a crucial component of both experimental colitis and IBD. The colons of mPGES-1^−/−^ mice exhibited significantly elevated mRNA levels of IL-17A (Th17 cytokine), as well as IFNγ (Th1 cytokine) and tumor necrosis factor α (TNFα), compared to the colons of WT mice on day 3 following TNBS administration (Figure 6). These findings suggest that mPGES-1-mediated PGE_2_ negatively regulates the excessive aberrant immunologic response linked to the Th17/Th1-related cytokines in colitis. Interestingly, the colonic expression of interleukin-1β (IL-1β), an important proinflammatory cytokine relevant to IBD, showed no differences between the two genotypes.

### 2.7. Generation of IL-17A- and IFNγ-Producing T Cells in mPGES-1^−/−^ Mice

**Figure 7:**
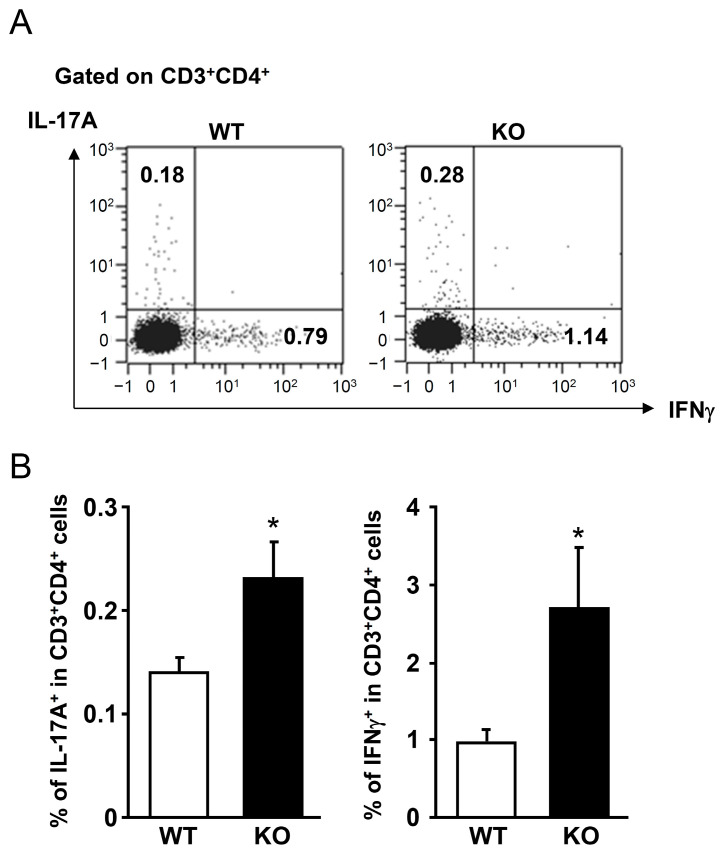
Population of IL-17A- and IFNγ-producing T cells in MLNs of mPGES-1^−/−^ mice. (**A**) Typical FCM data showing IL-17A-producing Th17 cells and IFNγ-producing Th1 cells in MLNs of WT and mPGES-1^−/−^ mice. On day 3 following the administration of TNBS, MLNs were isolated and assessed using FCM, as mentioned in the Materials and Methods. (**B**) Proportion of IL-17A^+^ and IFNγ^+^ cells in MLNs on day 3 following TNBS treatment (*n* = 6). *, *p* < 0.05 vs. WT; *t* test.

To investigate the role of mPGES-1 in the development of Th17/Th1 immune responses involved in colitis, we next analyzed the proportions of Th17 and Th1 cells that produced IL-17A and IFNγ in mesenteric lymph nodes (MLNs) of mPGES-1^−/−^ and WT mice following colitis induction. During the colitis phase, cells were harvested, cultured with phorbol 12-myristate 13-acetate and ionomycin, and subsequently stained for CD3/CD4, followed by fixation, intracellular staining for IL-17A and IFNγ, and analysis using flow cytometry (FCM). We observed a higher population of IL-17A-producing Th17 cells and IFNγ-producing Th1 cells in mPGES-1^−/−^ mice compared to WT mice, which is consistent with the enhanced colonic expression of Th17/Th1-related cytokines due to the absence of mPGES-1 (Figure 7).

## 3. Discussion

This study showed, for the first time, that mPGES-1 is crucial for the development of colitis, as evidenced by the severe inflammatory responses and histological characteristics observed following the deletion of the mPGES-1 gene. Additionally, our research revealed a significant enhancement of Th17 and Th1 immune responses in the context of mPGES-1 deficiency. These findings are consistent with previous reports. Pharmacological inhibition of COX-2, along with the resulting reduction in colonic PGE_2_ levels, has been shown to exacerbate NSAID-related TNBS-induced colitis in rat models [35]. Another study indicated significant alterations in the mRNA expression of the PGE_2_ receptor EP_4_ and PGE_2_ biosynthetic enzymes, including mPGES-1 and COX-2, in guinea pig and rat models of TNBS-induced ileitis [36]. Furthermore, it has been reported that EP_4_ signaling mitigates TNBS-induced colitis and reduces colonic mRNA expression of colitogenic cytokines, including IFNg [37]. In conjunction with previous studies, our findings suggest that the COX/mPGES-1/PGE_2_ pathway may be crucial in TNBS-induced colitis, primarily through EP_4_ signaling.

A lack of mPGES-1 exacerbated the severity of colitis, resulting in severe diarrhea, colon bleeding, epithelial damage, and inflammatory cell infiltration following the administration of TNBS. Therefore, mPGES-1 may play a protective role in TNBS-induced colitis. Notably, we did not observe any significant differences in body weight loss between WT and mPGES-1^−/−^ mice after TNBS administration. Concerning body weight monitoring as a major assessment of colitis, the mice exhibited an almost maximum decrease in body weight due to overnight fasting prior to the induction of colitis. This substantial loss of body weight may contribute to the fragility of the mice following TNBS administration.

A key finding of the present study is that the deficiency of mPGES-1 resulted in the upregulation of colonic COX-2 protein during colitis. This finding indicates that mPGES-1, along with its produced PGE_2_, modulates the induction of its upstream enzyme via a feedback loop. Our study also revealed that mPGES-1 plays an essential role in the generation of colonic PGE_2_. This is in agreement with observations of the responses in other tissues and cell types, including macrophages, dendritic cells, splenocytes, and embryonic fibroblasts [18,20,38,39,40].

It is noteworthy that we observed elevated levels of colonic PGD_2_ production in mPGES-1^−/−^ mice, which appeared to result from the diversion of PG precursors into PGD_2_ synthesis in the absence of mPGES-1. Additionally, we detected increased COX-2 protein levels due to the deletion of mPGES-1. These findings suggest that the increase in COX-2, along with the resulting enhancement in the availability of PGH_2_—as the common substrate—contributes to the elevated PGD_2_ production in mPGES-1^−/−^ mice. This conclusion is further supported by our recent research on DSS-induced colitis, which also showed a shift toward PGD_2_ production accompanied by upregulation of COX-2 expression in the context of mPGES-1 deficiency [25]. Since previous studies have shown the anti-colitis properties of PGD_2_ and its synthesizing enzymes in experimental colitis, as well as in human IBD [10,34,41,42], we propose that the elevated levels of PGD_2_ may alleviate colitis in the lack of mPGES-1. However, our findings reveal that the deletion of mPGES-1 conversely alleviates colitis, accompanied by a marked reduction in PGE_2_, despite an increase in the anti-colitis effects of PGD_2_. This result suggests that mPGES-1-driven PGE_2_ possesses more significant anti-colitis properties than PGD_2_ as a prostanoid. In the present study, we utilized ELISA to measure the levels of prostanoid; however, it may be important to employ a more specific method, such as LC-MS/MS, to further validate our findings.

We conducted further investigations into the role of mPGES-1 in pathogenic immunity in IBD using mice with TNBS-induced colitis. This study revealed that mPGES-1 deficiency enhances the expression of Th17/Th1 cytokines in response to TNBS. Additionally, we observed an increase in the population of IL-17A-producing Th17 cells in MLNs when mPGES-1 is absent, suggesting that Th17 differentiation and expansion during colitis is modulated by an mPGES-1-dependent mechanism. Our study also showed an increased population of colonic IFNγ-producing Th1 cells in MLNs. The facilitation of Th17 and Th1 immunity in mPGES-1^−/−^ mice may be linked to the abnormalities observed in TNBS-induced colitis.

We also verified that the absence of mPGES-1 leads to higher expression levels of TNFα in TNBS-induced colitis. In contrast, our study found that the differences in IL-1β expression levels in WT and mPGES-1^−/−^ mice during TNBS-induced colitis were not statistically significant. These findings are supported by our recent study using a DSS-induced colitis model, which showed similar expression profiles of TNFα and IL-1β in the absence of mPGES-1 during colitis [25].

Although we focused on the IFNγ-producing Th1 cells and IL-17A-producing Th17 cells in the present study, macrophages also play a crucial role in maintaining intestinal homeostasis and seem to be responsible for driving inflammation in IBD [43]. Macrophages release a variety of inflammatory mediators through multiple mechanisms. It has been reported that inflammatory macrophages strongly express key colitogenic mediators, such as inducible nitric oxide synthase (iNOS), which is regulated by the IFNγ-dependent epigenetic pathway [44]. The induction of iNOS in IBD involves both Th1 and Th17 cytokines. Additionally, findings from animal models and clinical studies suggest a positive association between elevated nitric oxide (NO) levels and colonic inflammation [45,46]. NO contributes to tissue damage and exacerbates inflammation indirectly by generating peroxynitrite [47,48]. We previously reported that iNOS expression and NO production are significantly elevated in mPGES-1^−/−^ embryonic fibroblasts compared to the low levels seen in WT embryonic fibroblasts, indicating that mPGES-1 deletion may influence not only the differential production of prostanoid in arachidonic acid metabolism but also the L-arginine metabolic pathway involving iNOS [18]. The detailed roles of mPGES-1/PGE_2_ in modulating the actions of macrophages associated with iNOS and its derived oxidants during colitis remain unclear.

It is remarkable that dysbiosis of the gut microbiome is increasingly linked to IBD [49]. In particular, short-chain fatty acids (SCFAs) derived from intestinal microbiota are crucial metabolites for maintaining intestinal homeostasis and are recognized for their role in enhancing gut barrier function. A previous study indicated that SCFAs promote the expression of epithelial mucin 2 by differentially influencing the production of PGE_2_ and PGE_1_ in intestinal myofibroblasts [50]. This finding may support our observation that mPGES-1 has a protective function against damage to the epithelial barrier. Additionally, SCFAs play significant roles in immunomodulation. They stimulate the release of PGE_2_ and the production of the anti-inflammatory cytokine IL-10, thereby reducing inflammatory responses in monocytes [51]. Although the precise molecular mechanisms by which SCFAs suppress pro-inflammatory mediators have not been fully elucidated, it is important to recognize that microbial metabolism of dietary fibers into SCFAs may modulate the intestinal immune system. Future studies should investigate the specific events related to the gut microbiome and microbiota-derived SCFAs associated with immune functions in the context of mPGES-1 deficiency.

## 4. Materials and Methods

### 4.1. Animals

mPGES-1^−/−^ mice with a C57BL/6 background were obtained from Oriental Bioservice Inc. (Kyoto, Japan) and were initially created by Prof. Shizuo Akir [17]. mPGES-1^−/−^ mice with a Balb/c background were produced by backcrossing at least 10 generations to Balb/c mice. A tail biopsy DNA extract was subjected to a polymerase chain reaction (PCR) using certain primers in order to identify genotypes [25]. The mice were kept in a specific pathogen-free barrier facility and were handled and cared for according to the protocols established by Kitasato University’s Safety Committee for Recombinant DNA Experiments and Animal Research and Ethics Committee. All animal studies were authorized by Kitasato University’s Animal Research and Ethics Committee (approval number Ei-ken 19-12), and all mPGES-1^−/−^ mice investigations were authorized by Kitasato University’s Safety Committee for Recombinant DNA investigations (approval number 4929).

### 4.2. Induction of Colitis

Male mice between the ages of 6 and 8 weeks were utilized in this study. Mice were fasted overnight prior to the procedure. Under isoflurane anesthesia, an enema (100 μL) of 2% TNBS (Sigma, St. Louis, MO, USA) in 40% ethanol was gradually injected into the colonic lumen using a 1 mL syringe attached to a flexible polytetrafluoroethylene sonde (Fuchigami, Kyoto, Japan), which had been inserted 3.8 cm from the anal verge [26]. For control animals, 100 μL of PBS was administered. To ensure thorough distribution of TNBS throughout the colon, mice were carefully kept at a 45-degree angle for 1 min before being returned to their cages. By measuring body weight loss and rating diarrhea and fecal bleeding on a scale of 0 (normal) to 4 (severe), the severity of colitis was evaluated every day. The DAI score was calculated as the sum of the diarrhea score and the fecal bleeding score, with a maximum possible score of 8 [25].

### 4.3. Histological Assessment of Colitis

Mice were euthanized under anesthesia on day 3 following TNBS administration, and their colons were harvested using the Swiss roll method. The colons were then fixed in 4% paraformaldehyde. Paraffin-embedded sections, each with a thickness of 3.5 μm, were subsequently stained with H&E. A blinded observer, unaware of the mice’s genotypes, conducted the histological assessment of colitis. The extent of epithelial disruption and the degree of inflammatory cell infiltration were utilized to assess the severity of colitis. The ratings for epithelial disruption were as follows: 0 for no visible damage, 1 for partial loss of goblet cells, 2 for significant loss of goblet cells, 3 for partial damage to the epithelial crypt, and 4 for complete damage to the crypt while the surface epithelium remains intact. Inflammatory cell infiltration was graded as follows: 0 for no infiltration, 1 for infiltration at the bases of the crypts, 2 for infiltration that reaches the muscularis mucosa, 3 for substantial infiltration that extends to the muscularis mucosa and causes thickening of the mucosa with significant edema, and 4 for infiltration of the submucosa. The histology score (maximum score: 8) was calculated by summing the scores for inflammatory cell infiltration and epithelial disruption [25,52,53].

### 4.4. Epithelial Barrier Permeability

FITC-dextran with an average molecular weight of 3000 to 5000 (FD4; Sigma, St Louis, MO, USA) was used to evaluate intestinal barrier function, according to previous reports [25,54]. In brief, mice were given FITC-dextran orally (10 mg/mouse at a dosage of 25 mg/mL) after being fasted overnight. Six hours later, blood was immediately harvested by heart puncture at the time of euthanasia under anesthesia. Using FLUOstar OPTIMA (BGM LABTECH, Offenburg, Germany) with excitation and emission wavelengths of 485 nm and 520 nm, respectively, the amount of FITC-dextran in serum was measured. A standard curve was generated using FITC-dextran dilutions.

### 4.5. Real-Time PCR Analysis

Total RNA was extracted using the NucleoSpin RNA kit (Macherey-Nagel, Duren, Germany), following a previous report [25]. After creating first-strand cDNAs using SuperScript VILO (Thermo Fisher Scientific, Waltham, MA, USA), real-time PCR was carried out in the MiniOpticon Real-Time PCR System (Bio-Rad, Hercules, CA, USA) using a Thunderbird SYBR qPCR Mix (Toyobo, Osaka, Japan). Table 1 lists the primer sets (Eurofins, Luxembourg City, Luxembourg) used in this study. The PCR cycling was conducted under the following conditions: 1 min at 95 °C, 40 cycles of 15 s each at 95 °C, and 1 min at 60 °C. Glyceraldehyde 3-phosphate dehydrogenase (GAPDH) was employed as a reference gene to normalize the threshold cycle values.

### 4.6. Western Blot Analysis

According to a previous study [25], the tissues were homogenized in a buffer consisting of 150 mmol/L, NaCl, 40 mmol/L Tris/HCl (pH 7.4), 2 mmol/L EDTA, 1 mmol/L dithiothreitol, 2 mmol/L sodium orthovanadate, 10 mmol/L sodium pyrophosphate, 10 mmol/L NaF, and 1% Triton X-100, along with a protease inhibitor cocktail (Sigma, St Louis, MO, USA). Bovine serum albumin was utilized as a reference, and protein concentrations were determined using a BCA protein assay kit (Thermo Fisher Scientific, Waltham, MA, USA). Using sodium dodecyl sulfate polyacrylamide gel electrophoresis, samples were separated, and proteins were then transferred to a PVDF membrane (GE Healthcare, Little Chalfont, UK). The membrane was blocked and then exposed to either anti-mPGES-1 (1:500 dilution, No. 160140; Cayman Chemicals, Ann Arbor, MI, USA), anti-cPGES (1:500 dilution, No. 160150; Cayman Chemicals, Ann Arbor, MI, USA), anti-COX-2 (1:500 dilution, No. 160106; Cayman Chemicals, Ann Arbor, MI, USA), anti-COX-1 (1:500 dilution, No. 160109; Cayman Chemicals, Ann Arbor, MI, USA), anti-hPGDS (1:500 dilution, No. 10004348; Cayman Chemicals, Ann Arbor, MI, USA), or anti-β-actin (1:10,000 dilution, A5441; Sigma, St Louis, MO, USA) antibody. Following the washing procedure, enhanced chemiluminescence (GE Healthcare, Little Chalfont, UK) was used to detect protein bands.

### 4.7. Measurement of PGE_2_ and PGD_2_

After homogenizing tissues in 70% methanol containing 30 μM indometacin, the tissue homogenates were centrifuged at 15,000× *g* for 20 min at 4 °C. The supernatant was evaporated using a nitrogen gas stream and then reconstituted in enzyme immunoassay buffer [25]. The concentrations of PGE_2_ and PGD_2_ as a MOX-PGD_2_ (a stable metabolite of PGD_2_) were then measured using enzyme-linked immunosorbent assay kits (Cayman Chemicals, Ann Arbor, MI, USA), following the manufacturer’s instructions. The Benchmark microplate reader (Bio-Rad, Hercules, CA, USA) was used to measure optical density.

### 4.8. FCM Analysis

Three days following the administration of TNBS, MLNs were isolated. Before labeling cell surface markers, single-cell suspensions of MLNs were treated with anti-CD16/32 antibody (TruStain fcX; BioLegend, San Diego, CA, USA) to prevent nonspecific antibody binding mediated by the FcγII/III receptor. Following intracellular staining for IFNγ and IL-17A, cells were labeled with fluorochrome-conjugated anti-mouse monoclonal antibodies (BioLegend, San Diego, CA, USA) against CD4 (clone GK1.5) and CD3 (clone 17A2). The background signal from off-target antibody binding was assessed using isotype controls. To eliminate dead cells and prevent background or non-specific staining of dead cells, the Zombie Aqua Fixable Viability kit (BioLegend, San Diego, CA, USA) was utilized. In order to identify T cells that produce IL-17A and IFNγ, intracellular staining for IL-17A (TC11-18H10.1; Biolegend, San Diego, CA, USA) and IFNγ (clones XMG1.2; Biolegend, San Diego, CA) was carried out following cell stimulation, surface molecule labeling, and cell fixation and permeabilization. As previously reported [22], cells were stimulated with phorbol 12-myristate 13-acetate (50 ng/mL, Sigma, St Louis, MO, USA), ionomycin (500 ng/mL, Sigma, St Louis, MO, USA), and GolgiStop (BD PharMingen, Franklin Lakes, NJ, USA) for 4 h in vitro in RPMI1640 supplemented with 10% fetal bovine serum, penicillin/streptomycin, and freshly added 50 μmol/L 2-mercaptoethanol. According to the manufacturer’s instructions, cells were fixed, permeabilized, and stained using the Cytofix/Cytoperm Plus Fixation/Permeabilization kit (BD PharMingen, Franklin Lakes, NJ, USA). Using a MACS Quant Analyzer (Miltenyi Biotec, Bergisch Gladbach, Germany), the stained cells were examined. The following hierarchy was always followed by the gating strategy: total events−lymphocyte gate (FSC-A/SSC-A)−live cells (Live/Dead−)−CD3^+^CD4^+^, with subsequent gating indicated in each experiment.

### 4.9. Statistical Analysis

Data are expressed as means ± SEM. Statistical analysis was conducted using Sigmastat version 3.5 (Systat Software, Inc., San Jose, CA, USA). After confirming a normal distribution, the t test was used to compare two groups, while analysis of variance (ANOVA) and the Bonferroni test were employed to analyze data from more than two groups. Statistical significance was defined as a *p*-value of below 0.05.

## 5. Conclusions

This study clearly indicates that mPGES-1 is the primary PGE synthase responsible for the production of intestinal PGE_2_, and that mPGES-1-driven PGE_2_ has a protective effect in IBD. mPGES-1 represents a potential target for drug development, as inhibiting mPGES-1 could specifically control the excessive production of PGE_2_ linked to a variety of inflammatory diseases. Nonetheless, the present study provides significant insights into the possible detrimental impact of pharmacological mPGES-1 inhibition in IBD.

## Figures and Tables

**Table 1:** Primer sequences of various target genes for real-time PCR.

Target Gene	Sense Primer	Antisense Primer	Accession No.
mPGES-1	5′-AGCACACTGCTGGTCATCAA-3′	5′-CTCCACATCTGGGTCACTCC-3′	AB041997
cPGES	5′-TGTTTGCGAAAAGGAGAATCCG-3′	5′-ACCCATGTGATCCATCATCTCA-3′	BC085264
COX-2	5′-AGGACTCTGCTCACGAAGGA-3′	5′-TGACATGGATTGGAACAGCA-3	NM_011198
COX-1	5′-GCCAGAACCAGGGTGTCTGT-3′	5′-GTAGCCCGTGCGAGTACAATC-3′	AK046457
IL-17A	5′-CAGGGAGAGCTTCATCTGTGT-3′	5′-GCTGAGCTTTGAGGGATGAT-3′	U43088
IFNg	5′-CGGCACAGTCATTGAAAGCCTA-3′	5′-GTTGCTGATGGCCTGATTGTC-3′	NM_008337
TNFα	5′-TCCCCAAAGGGATGAGAAG-3′	5′-CACTTGGTGGTTTGCTACGA-3′	NM_013693
IL-1β	5′-ACTGTGAAATGCCACCTTTTG-3′	5′-TGTTGATGTGCTGCTGCGAG-3′	NM_008361
GAPDH	5′-GTCTTCACCACCATGGAGAAGG-3′	5′-TCATGGATGACCTTGGCCAG-3′	GU214026

## Data Availability

Data are contained within this article.
